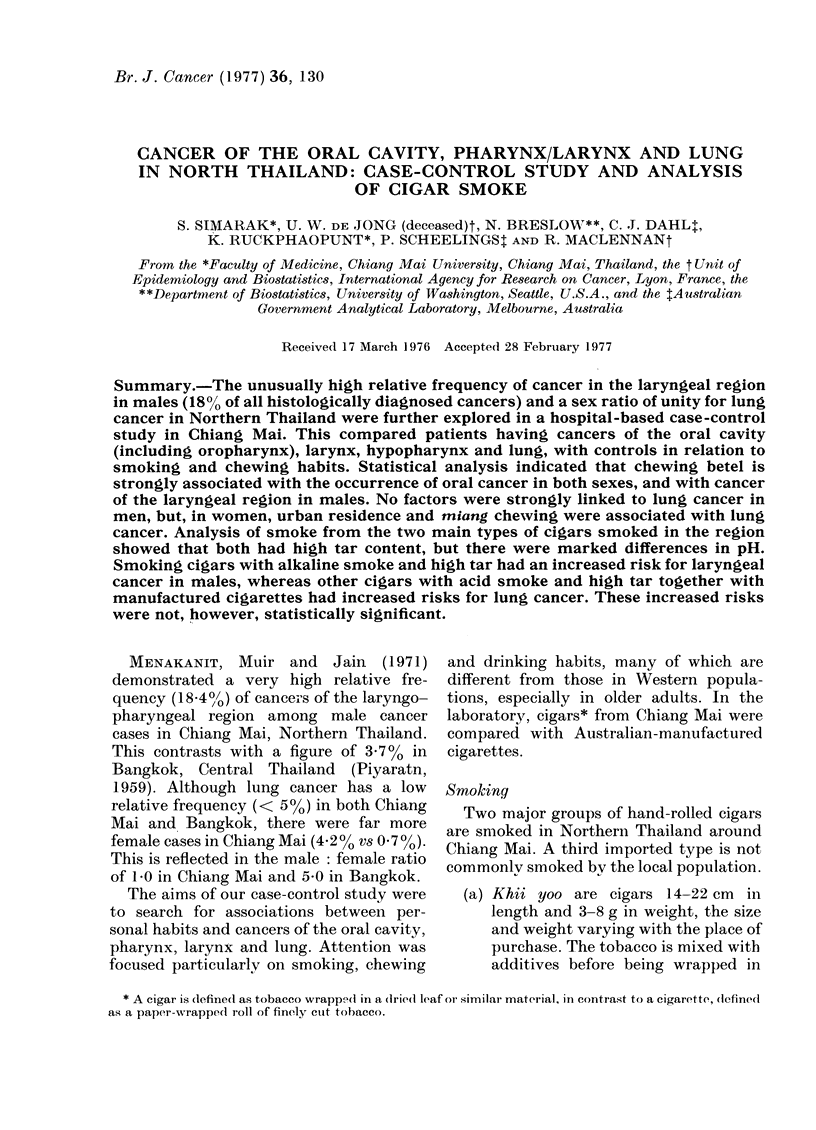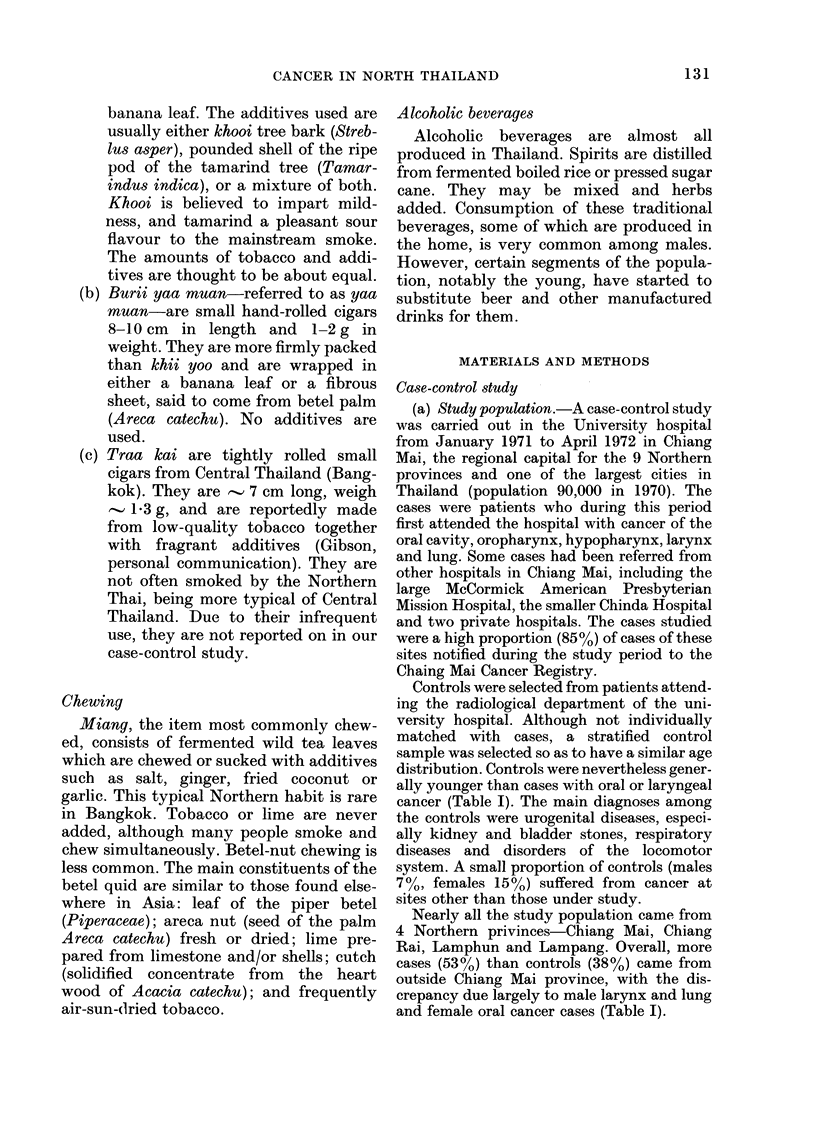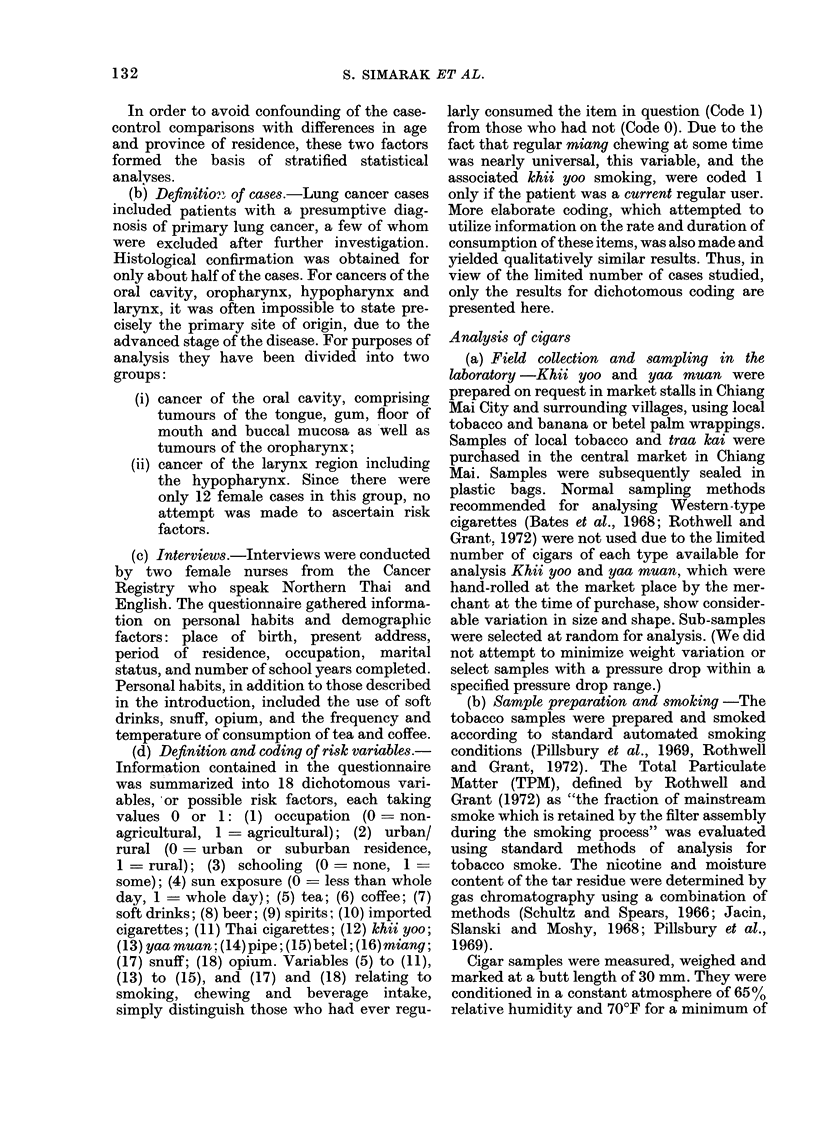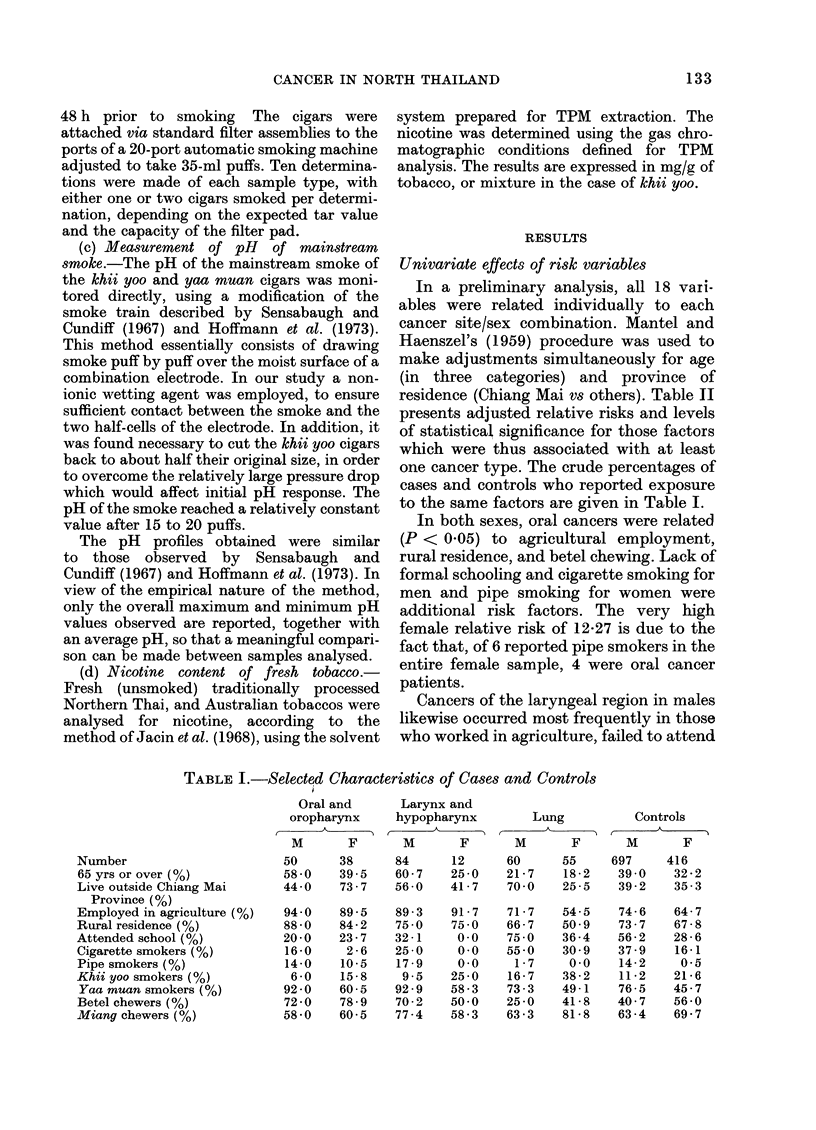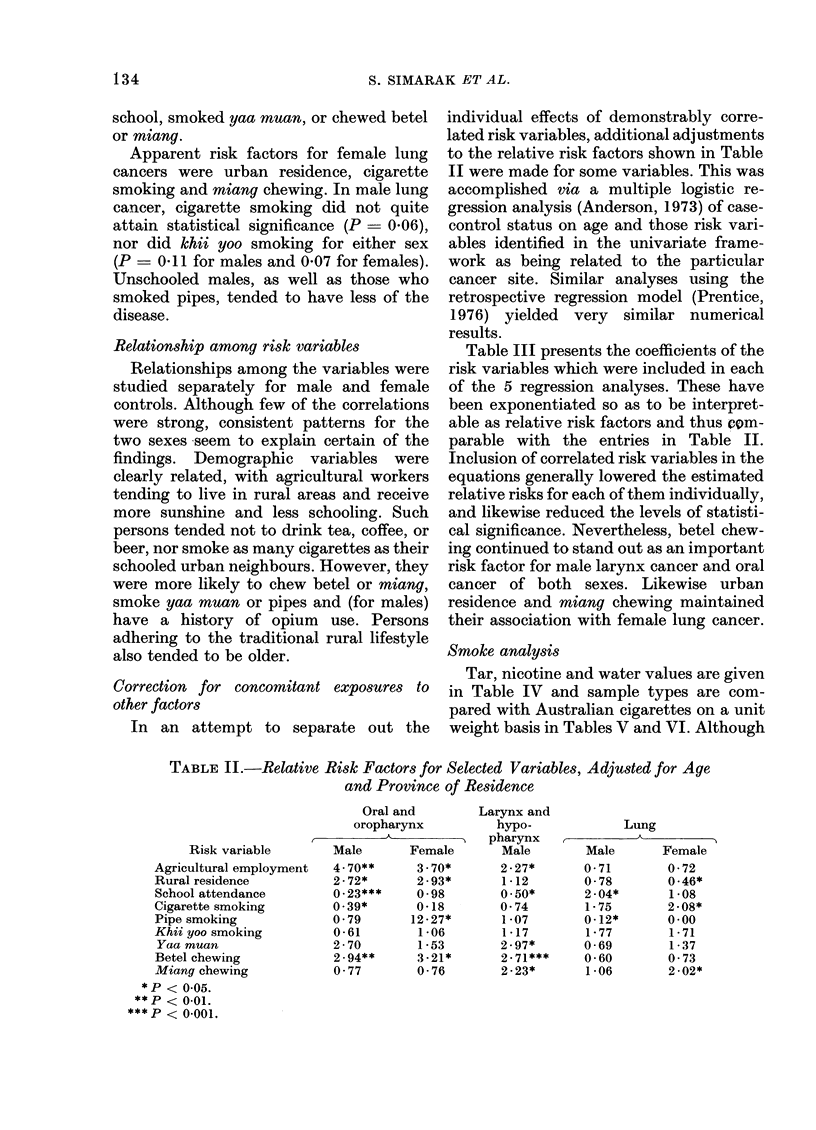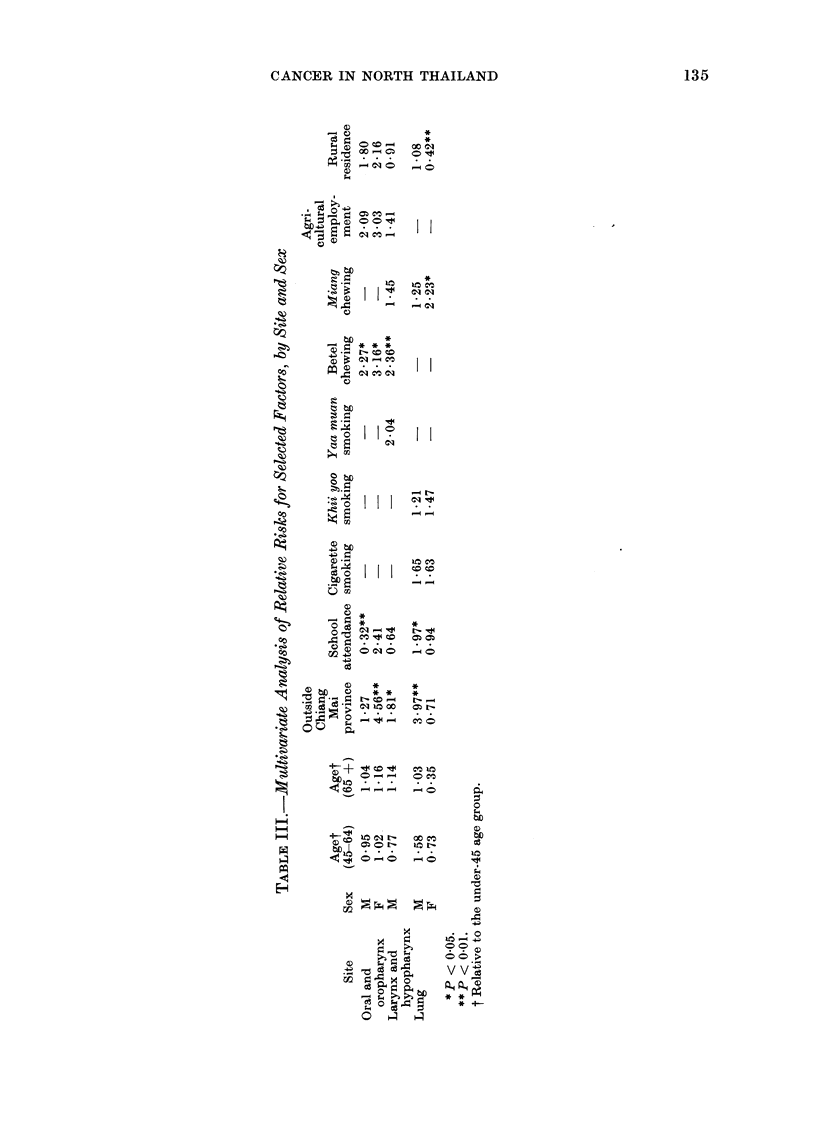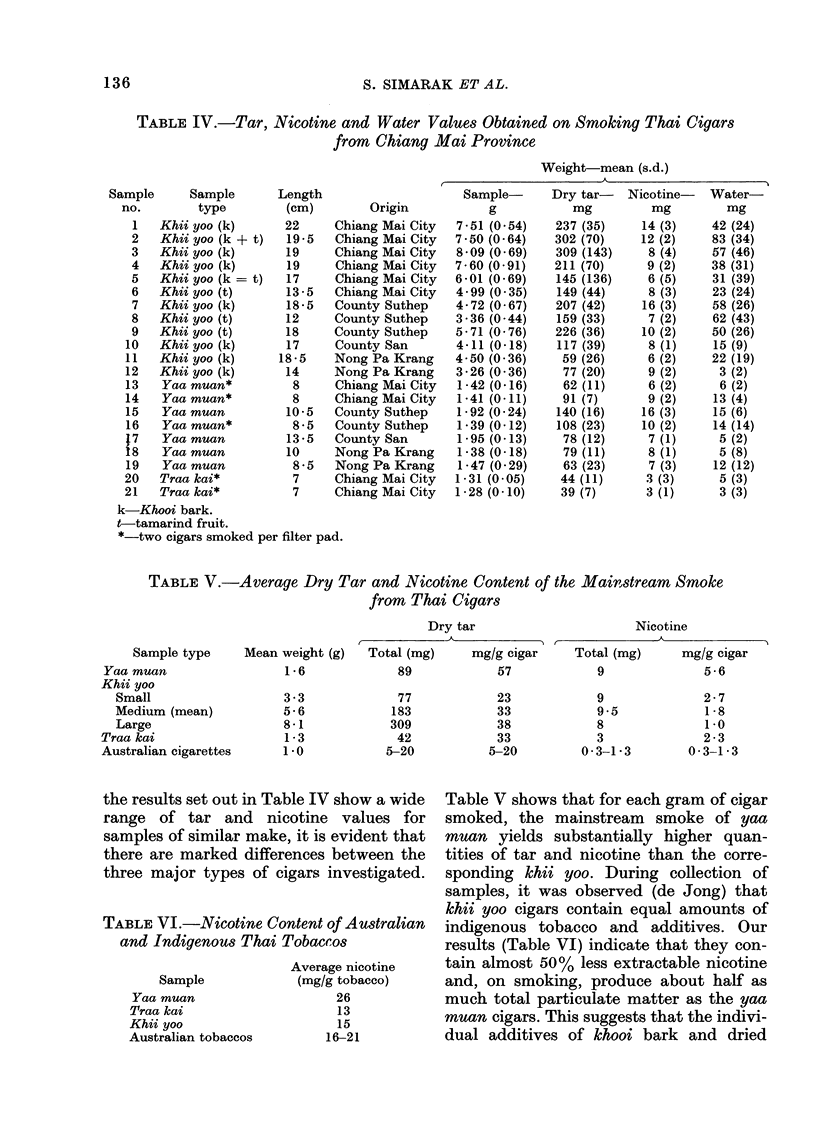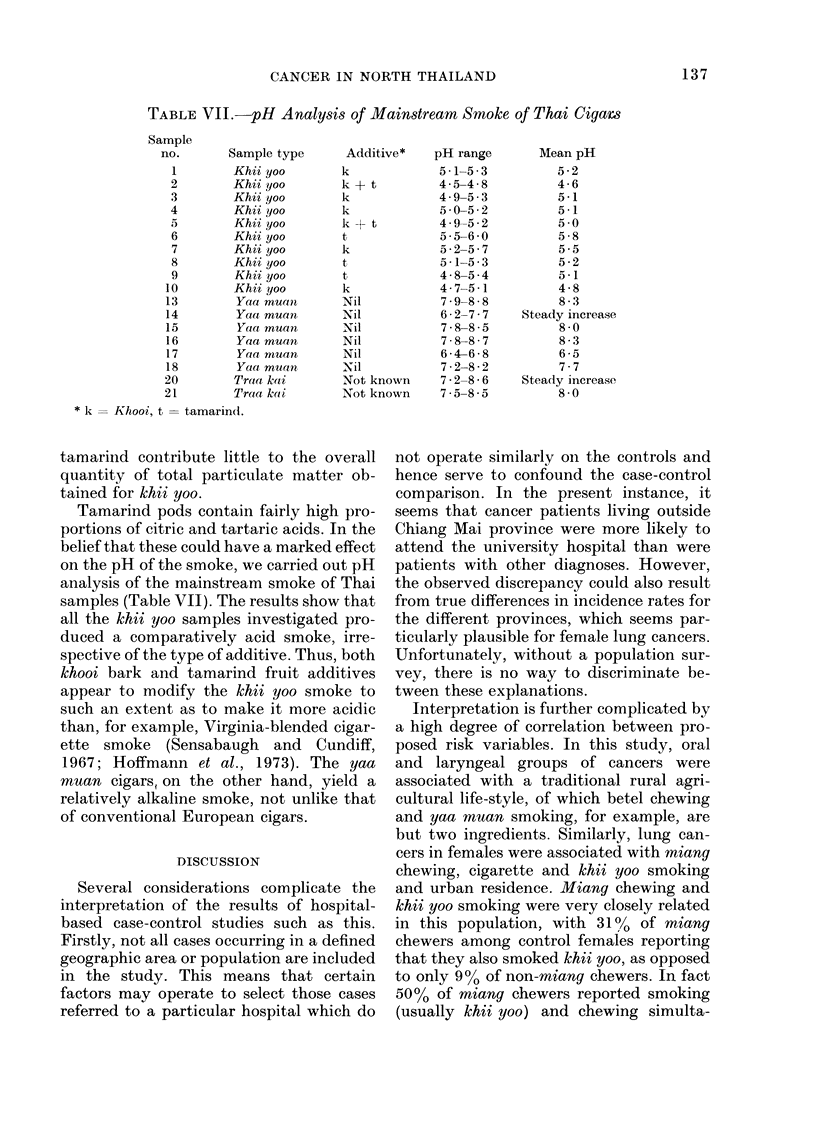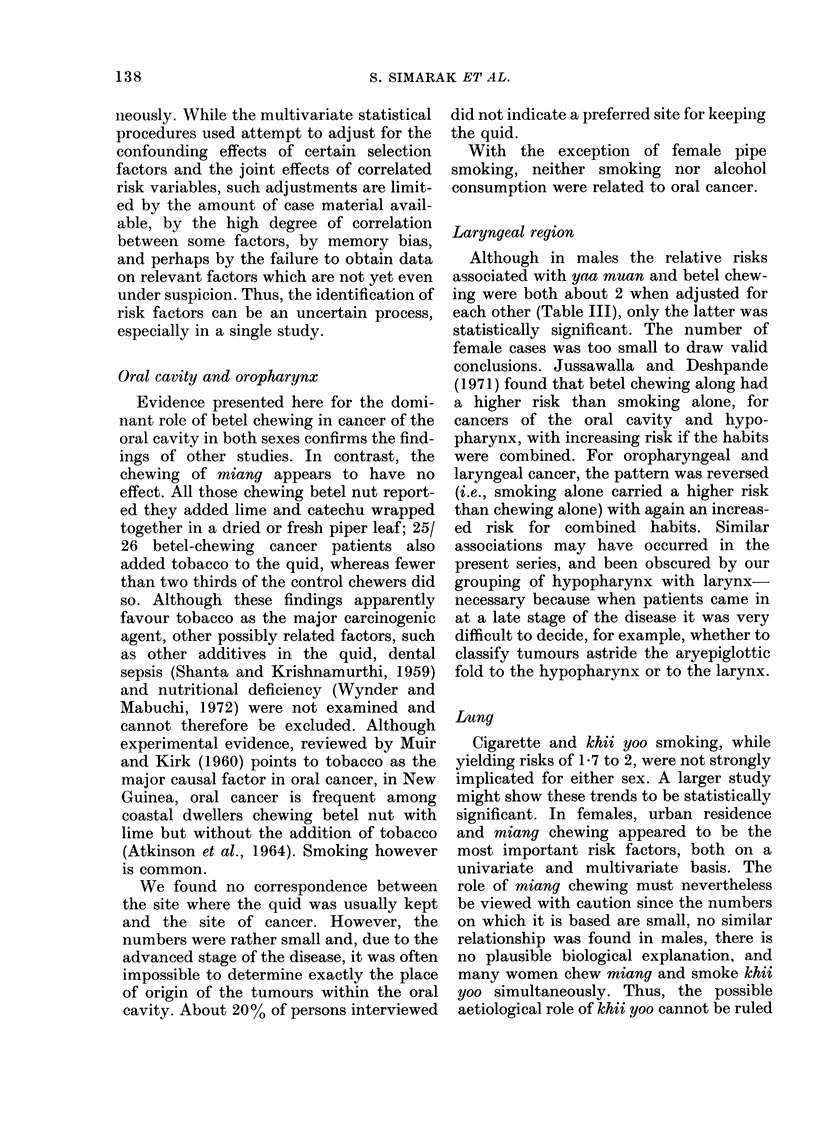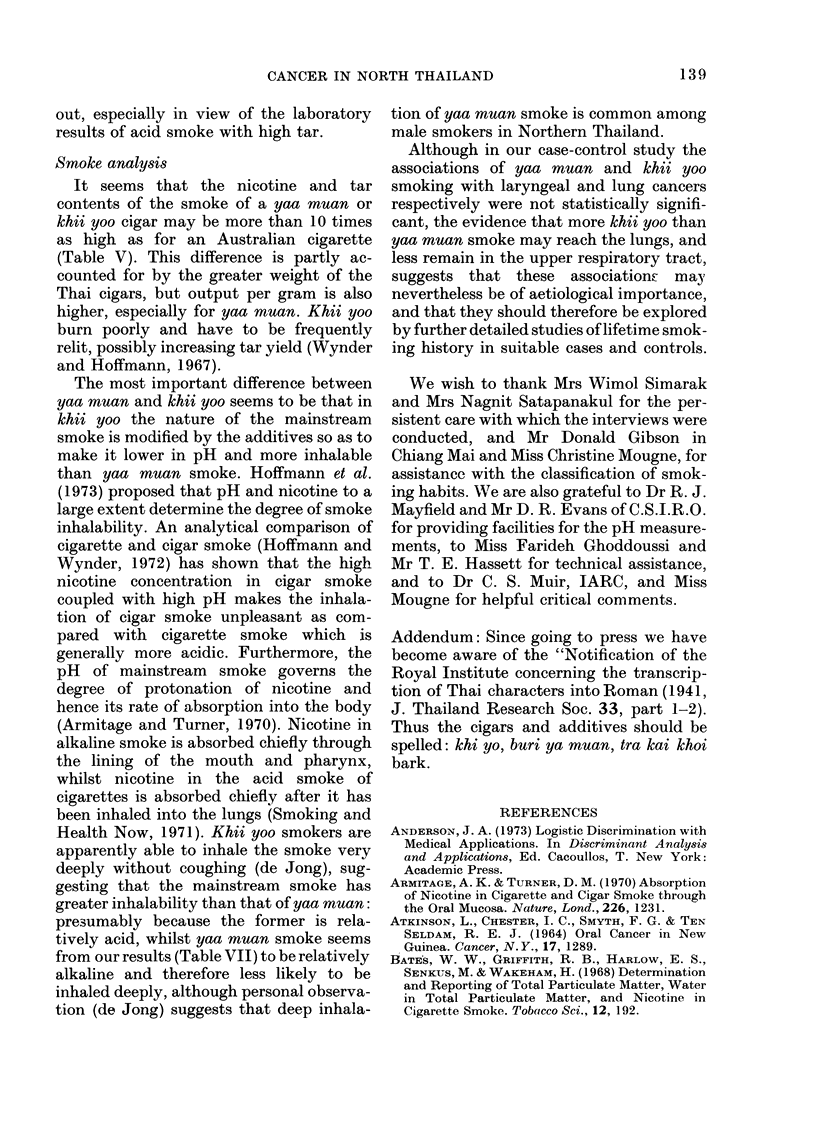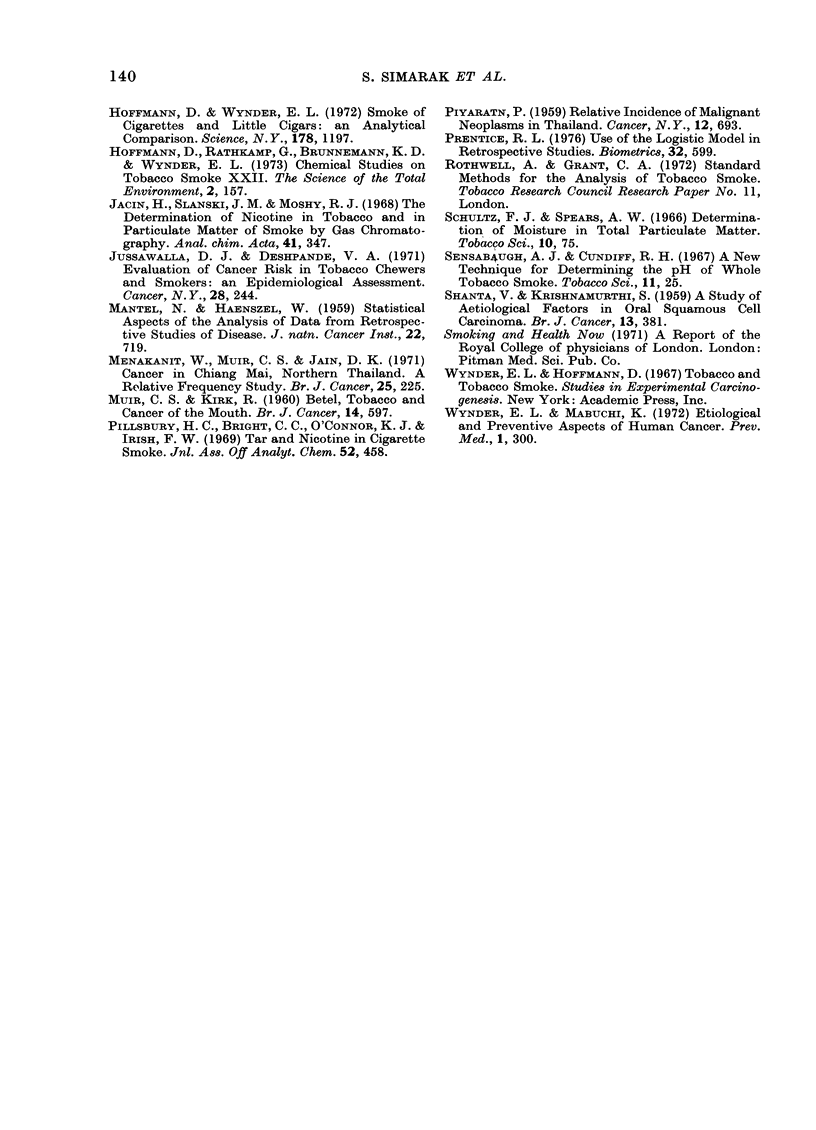# Cancer of the oral cavity, pharynx/larynx and lung in North Thailand: case-control study and analysis of cigar smoke.

**DOI:** 10.1038/bjc.1977.163

**Published:** 1977-07

**Authors:** S. Simarak, U. W. de Jong, N. Breslow, C. J. Dahl, K. Ruckphaopunt, P. Scheelings, R. Maclennan

## Abstract

The unusually high relative frequency of cancer in the laryngeal region in males (18% of all histologically diagnosed cancers) and a sex ratio of unity for lung cancer in Northern Thailand were further explored in a hospital-based case-control study in Chiang Mai. This compared patients having cancers of the oral cavity (including oropharynx), larynx, hypopharynx and lung, with controls in relation to smoking and chewing habits. Statistical analysis indicated that chewing betel is strongly associated with the occurrence of oral cancer in both sexes, and with cancer of the laryngeal region in males. No factors were strongly linked to lung cancer in men, but, in women, urban residence and miang chewing were associated with lung cancer. Analysis of smoke from the two main types of cigars smoked in the region showed that both had high tar content, but there were marked differences in pH. Smoking cigars with alkaline smoke and high tar had an increased risk for laryngeal cancer in males, whereas other cigars with acid smoke and high tar together with manufactured cigarettes had increased risks for lung cancer. These increased risks were not, however, statistically significant.


*Br. J. Cancer* (1977) **36**, 130

### CANCER OF THE ORAL CAVITY, PHARYNX/LARYNX AND LUNG IN NORTH THAILAND: CASE-CONTROL STUDY AND ANALYSIS OF CIGAR SMOKE

S. SIMARAK*, U. W. DE JONG (deceased)†, N. BRESLOW**, C. J. DAHL‡, K. RUCKPHAOPUNT*, P. SCHEELINGS‡ AND R. MACLENNAN†

*From the *Faculty of Medicine, Chiang Mai University, Chiang Mai, Thailand, the †Unit of Epidemiology and Biostatistics, International Agency for Research on Cancer, Lyon, France, the **Department of Biostatistics, University of Washington, Seattle, U.S.A., and the ‡Australian Government Analytical Laboratory, Melbourne, Australia*

Received 17 March 1976 Accepted 28 February 1977

**Summary.—The unusually high relative frequency of cancer in the laryngeal region in males (18% of all histologically diagnosed cancers) and a sex ratio of unity for lung cancer in Northern Thailand were further explored in a hospital-based case-control study in Chiang Mai. This compared patients having cancers of the oral cavity (including oropharynx), larynx, hypopharynx and lung, with controls in relation to smoking and chewing habits. Statistical analysis indicated that chewing betel is strongly associated with the occurrence of oral cancer in both sexes, and with cancer of the laryngeal region in males. No factors were strongly linked to lung cancer in men, but, in women, urban residence and *miang* chewing were associated with lung cancer. Analysis of smoke from the two main types of cigars smoked in the region showed that both had high tar content, but there were marked differences in pH. Smoking cigars with alkaline smoke and high tar had an increased risk for laryngeal cancer in males, whereas other cigars with acid smoke and high tar together with manufactured cigarettes had increased risks for lung cancer. These increased risks were not, however, statistically significant.**

MENAKANIT, Muir and Jain (1971) demonstrated a very high relative frequency (18·4%) of cancers of the laryngo-pharyngeal region among male cancer cases in Chiang Mai, Northern Thailand. This contrasts with a figure of 3·7% in Bangkok, Central Thailand (Piyaratn, 1959). Although lung cancer has a low relative frequency (< 5%) in both Chiang Mai and Bangkok, there were far more female cases in Chiang Mai (4·2% *vs* 0·7%). This is reflected in the male : female ratio of 1·0 in Chiang Mai and 5·0 in Bangkok.

The aims of our case-control study were to search for associations between personal habits and cancers of the oral cavity, pharynx, larynx and lung. Attention was focused particularly on smoking, chewing and drinking habits, many of which are different from those in Western populations, especially in older adults. In the laboratory, cigars* from Chiang Mai were compared with Australian-manufactured cigarettes.

*Smoking*

Two major groups of hand-rolled cigars are smoked in Northern Thailand around Chiang Mai. A third imported type is not commonly smoked by the local population.

(a) *Khii yoo* are cigars 14–22 cm in length and 3–8 g in weight, the size and weight varying with the place of purchase. The tobacco is mixed with additives before being wrapped in

* A cigar is defined as tobacco wrapped in a dried leaf or similar material, in contrast to a cigarette, defined as a paper-wrapped roll of finely cut tobacco.

banana leaf. The additives used are usually either *khooi* tree bark (*Streblus asper*), pounded shell of the ripe pod of the tamarind tree (*Tamarindus indica*), or a mixture of both. *Khooi* is believed to impart mildness, and tamarind a pleasant sour flavour to the mainstream smoke. The amounts of tobacco and additives are thought to be about equal.

(b) *Burii yaa muan*—referred to as *yaa muan*—are small hand-rolled cigars 8–10 cm in length and 1–2 g in weight. They are more firmly packed than *khii yoo* and are wrapped in either a banana leaf or a fibrous sheet, said to come from betel palm (*Areca catechu*). No additives are used.

(c) *Traa kai* are tightly rolled small cigars from Central Thailand (Bangkok). They are $\sim$ 7 cm long, weigh $\sim$ 1·3 g, and are reportedly made from low-quality tobacco together with fragrant additives (Gibson, personal communication). They are not often smoked by the Northern Thai, being more typical of Central Thailand. Due to their infrequent use, they are not reported on in our case-control study.

*Chewing*

*Miang*, the item most commonly chewed, consists of fermented wild tea leaves which are chewed or sucked with additives such as salt, ginger, fried coconut or garlic. This typical Northern habit is rare in Bangkok. Tobacco or lime are never added, although many people smoke and chew simultaneously. Betel-nut chewing is less common. The main constituents of the betel quid are similar to those found elsewhere in Asia: leaf of the piper betel (*Piperaceae*); areca nut (seed of the palm *Areca catechu*) fresh or dried; lime prepared from limestone and/or shells; cutch (solidified concentrate from the heart wood of *Acacia catechu*); and frequently air-sun-dried tobacco.

*Alcoholic beverages*

Alcoholic beverages are almost all produced in Thailand. Spirits are distilled from fermented boiled rice or pressed sugar cane. They may be mixed and herbs added. Consumption of these traditional beverages, some of which are produced in the home, is very common among males. However, certain segments of the population, notably the young, have started to substitute beer and other manufactured drinks for them.

### MATERIALS AND METHODS

*Case-control study*

(a) *Study population.*—A case-control study was carried out in the University hospital from January 1971 to April 1972 in Chiang Mai, the regional capital for the 9 Northern provinces and one of the largest cities in Thailand (population 90,000 in 1970). The cases were patients who during this period first attended the hospital with cancer of the oral cavity, oropharynx, hypopharynx, larynx and lung. Some cases had been referred from other hospitals in Chiang Mai, including the large McCormick American Presbyterian Mission Hospital, the smaller Chinda Hospital and two private hospitals. The cases studied were a high proportion (85%) of cases of these sites notified during the study period to the Chaing Mai Cancer Registry.

Controls were selected from patients attending the radiological department of the university hospital. Although not individually matched with cases, a stratified control sample was selected so as to have a similar age distribution. Controls were nevertheless generally younger than cases with oral or laryngeal cancer (Table I). The main diagnoses among the controls were urogenital diseases, especially kidney and bladder stones, respiratory diseases and disorders of the locomotor system. A small proportion of controls (males 7%, females 15%) suffered from cancer at sites other than those under study.

Nearly all the study population came from 4 Northern privinces—Chiang Mai, Chiang Rai, Lamphun and Lampang. Overall, more cases (53%) than controls (38%) came from outside Chiang Mai province, with the discrepancy due largely to male larynx and lung and female oral cancer cases (Table I).

In order to avoid confounding of the case-control comparisons with differences in age and province of residence, these two factors formed the basis of stratified statistical analyses.

(b) *Definition of cases.*—Lung cancer cases included patients with a presumptive diagnosis of primary lung cancer, a few of whom were excluded after further investigation. Histological confirmation was obtained for only about half of the cases. For cancers of the oral cavity, oropharynx, hypopharynx and larynx, it was often impossible to state precisely the primary site of origin, due to the advanced stage of the disease. For purposes of analysis they have been divided into two groups:

(i) cancer of the oral cavity, comprising tumours of the tongue, gum, floor of mouth and buccal mucosa as well as tumours of the oropharynx;

(ii) cancer of the larynx region including the hypopharynx. Since there were only 12 female cases in this group, no attempt was made to ascertain risk factors.

(c) *Interviews.*—Interviews were conducted by two female nurses from the Cancer Registry who speak Northern Thai and English. The questionnaire gathered information on personal habits and demographic factors: place of birth, present address, period of residence, occupation, marital status, and number of school years completed. Personal habits, in addition to those described in the introduction, included the use of soft drinks, snuff, opium, and the frequency and temperature of consumption of tea and coffee.

(d) *Definition and coding of risk variables.*—Information contained in the questionnaire was summarized into 18 dichotomous variables, or possible risk factors, each taking values 0 or 1: (1) occupation (0 = non-agricultural, 1 = agricultural); (2) urban/rural (0 = urban or suburban residence, 1 = rural); (3) schooling (0 = none, 1 = some); (4) sun exposure (0 = less than whole day, 1 = whole day); (5) tea; (6) coffee; (7) soft drinks; (8) beer; (9) spirits; (10) imported cigarettes; (11) Thai cigarettes; (12) *khii yoo*; (13) *yaa muan*; (14) pipe; (15) betel; (16) *miang*; (17) snuff; (18) opium. Variables (5) to (11), (13) to (15), and (17) and (18) relating to smoking, chewing and beverage intake, simply distinguish those who had ever regularly consumed the item in question (Code 1) from those who had not (Code 0). Due to the fact that regular *miang* chewing at some time was nearly universal, this variable, and the associated *khii yoo* smoking, were coded 1 only if the patient was a *current* regular user. More elaborate coding, which attempted to utilize information on the rate and duration of consumption of these items, was also made and yielded qualitatively similar results. Thus, in view of the limited number of cases studied, only the results for dichotomous coding are presented here.

*Analysis of cigars*

(a) *Field collection and sampling in the laboratory*—*Khii yoo* and *yaa muan* were prepared on request in market stalls in Chiang Mai City and surrounding villages, using local tobacco and banana or betel palm wrappings. Samples of local tobacco and *traa kai* were purchased in the central market in Chiang Mai. Samples were subsequently sealed in plastic bags. Normal sampling methods recommended for analysing Western-type cigarettes (Bates *et al.*, 1968; Rothwell and Grant, 1972) were not used due to the limited number of cigars of each type available for analysis *Khii yoo* and *yaa muan*, which were hand-rolled at the market place by the merchant at the time of purchase, show considerable variation in size and shape. Sub-samples were selected at random for analysis. (We did not attempt to minimize weight variation or select samples with a pressure drop within a specified pressure drop range.)

(b) *Sample preparation and smoking*—The tobacco samples were prepared and smoked according to standard automated smoking conditions (Pillsbury *et al.*, 1969, Rothwell and Grant, 1972). The Total Particulate Matter (TPM), defined by Rothwell and Grant (1972) as "the fraction of mainstream smoke which is retained by the filter assembly during the smoking process" was evaluated using standard methods of analysis for tobacco smoke. The nicotine and moisture content of the tar residue were determined by gas chromatography using a combination of methods (Schultz and Spears, 1966; Jacin, Slanski and Moshy, 1968; Pillsbury *et al.*, 1969).

Cigar samples were measured, weighed and marked at a butt length of 30 mm. They were conditioned in a constant atmosphere of 65% relative humidity and 70°F for a minimum of

48 h prior to smoking The cigars were attached *via* standard filter assemblies to the ports of a 20-port automatic smoking machine adjusted to take 35-ml puffs. Ten determinations were made of each sample type, with either one or two cigars smoked per determination, depending on the expected tar value and the capacity of the filter pad.

(c) *Measurement of pH of mainstream smoke.*—The pH of the mainstream smoke of the *khii yoo* and *yaa muan* cigars was monitored directly, using a modification of the smoke train described by Sensabaugh and Cundiff (1967) and Hoffmann *et al.* (1973). This method essentially consists of drawing smoke puff by puff over the moist surface of a combination electrode. In our study a non-ionic wetting agent was employed, to ensure sufficient contact between the smoke and the two half-cells of the electrode. In addition, it was found necessary to cut the *khii yoo* cigars back to about half their original size, in order to overcome the relatively large pressure drop which would affect initial pH response. The pH of the smoke reached a relatively constant value after 15 to 20 puffs.

The pH profiles obtained were similar to those observed by Sensabaugh and Cundiff (1967) and Hoffmann *et al.* (1973). In view of the empirical nature of the method, only the overall maximum and minimum pH values observed are reported, together with an average pH, so that a meaningful comparison can be made between samples analysed.

(d) *Nicotine content of fresh tobacco.*—Fresh (unsmoked) traditionally processed Northern Thai, and Australian tobaccos were analysed for nicotine, according to the method of Jacin *et al.* (1968), using the solvent system prepared for TPM extraction. The nicotine was determined using the gas chromatographic conditions defined for TPM analysis. The results are expressed in mg/g of tobacco, or mixture in the case of *khii yoo*.

### RESULTS

### *Univariate effects of risk variables*

In a preliminary analysis, all 18 variables were related individually to each cancer site/sex combination. Mantel and Haenszel's (1959) procedure was used to make adjustments simultaneously for age (in three categories) and province of residence (Chiang Mai *vs* others). Table II presents adjusted relative risks and levels of statistical significance for those factors which were thus associated with at least one cancer type. The crude percentages of cases and controls who reported exposure to the same factors are given in Table I.

In both sexes, oral cancers were related ($P < 0{\cdot}05$) to agricultural employment, rural residence, and betel chewing. Lack of formal schooling and cigarette smoking for men and pipe smoking for women were additional risk factors. The very high female relative risk of 12·27 is due to the fact that, of 6 reported pipe smokers in the entire female sample, 4 were oral cancer patients.

Cancers of the laryngeal region in males likewise occurred most frequently in those who worked in agriculture, failed to attend

TABLE I.—*Selected Characteristics of Cases and Controls*

| | Oral and oropharynx | | Larynx and hypopharynx | | Lung | | Controls | |
|---|---|---|---|---|---|---|---|---|
| | M | F | M | F | M | F | M | F |
| Number | 50 | 38 | 84 | 12 | 60 | 55 | 697 | 416 |
| 65 yrs or over (%) | 58·0 | 39·5 | 60·7 | 25·0 | 21·7 | 18·2 | 39·0 | 32·2 |
| Live outside Chiang Mai Province (%) | 44·0 | 73·7 | 56·0 | 41·7 | 70·0 | 25·5 | 39·2 | 35·3 |
| Employed in agriculture (%) | 94·0 | 89·5 | 89·3 | 91·7 | 71·7 | 54·5 | 74·6 | 64·7 |
| Rural residence (%) | 88·0 | 84·2 | 75·0 | 75·0 | 66·7 | 50·9 | 73·7 | 67·8 |
| Attended school (%) | 20·0 | 23·7 | 32·1 | 0·0 | 75·0 | 36·4 | 56·2 | 28·6 |
| Cigarette smokers (%) | 16·0 | 2·6 | 25·0 | 0·0 | 55·0 | 30·9 | 37·9 | 16·1 |
| Pipe smokers (%) | 14·0 | 10·5 | 17·9 | 0·0 | 1·7 | 0·0 | 14·2 | 0·5 |
| *Khii yoo* smokers (%) | 6·0 | 15·8 | 9·5 | 25·0 | 16·7 | 38·2 | 11·2 | 21·6 |
| *Yaa muan* smokers (%) | 92·0 | 60·5 | 92·9 | 58·3 | 73·3 | 49·1 | 76·5 | 45·7 |
| Betel chewers (%) | 72·0 | 78·9 | 70·2 | 50·0 | 25·0 | 41·8 | 40·7 | 56·0 |
| *Miang* chewers (%) | 58·0 | 60·5 | 77·4 | 58·3 | 63·3 | 81·8 | 63·4 | 69·7 |

school, smoked *yaa muan*, or chewed betel or *miang*.

Apparent risk factors for female lung cancers were urban residence, cigarette smoking and *miang* chewing. In male lung cancer, cigarette smoking did not quite attain statistical significance ($P = 0{\cdot}06$), nor did *khii yoo* smoking for either sex ($P = 0{\cdot}11$ for males and 0·07 for females). Unschooled males, as well as those who smoked pipes, tended to have less of the disease.

### *Relationship among risk variables*

Relationships among the variables were studied separately for male and female controls. Although few of the correlations were strong, consistent patterns for the two sexes seem to explain certain of the findings. Demographic variables were clearly related, with agricultural workers tending to live in rural areas and receive more sunshine and less schooling. Such persons tended not to drink tea, coffee, or beer, nor smoke as many cigarettes as their schooled urban neighbours. However, they were more likely to chew betel or *miang*, smoke *yaa muan* or pipes and (for males) have a history of opium use. Persons adhering to the traditional rural lifestyle also tended to be older.

### *Correction for concomitant exposures to other factors*

In an attempt to separate out the individual effects of demonstrably correlated risk variables, additional adjustments to the relative risk factors shown in Table II were made for some variables. This was accomplished *via* a multiple logistic regression analysis (Anderson, 1973) of case-control status on age and those risk variables identified in the univariate framework as being related to the particular cancer site. Similar analyses using the retrospective regression model (Prentice, 1976) yielded very similar numerical results.

Table III presents the coefficients of the risk variables which were included in each of the 5 regression analyses. These have been exponentiated so as to be interpretable as relative risk factors and thus comparable with the entries in Table II. Inclusion of correlated risk variables in the equations generally lowered the estimated relative risks for each of them individually, and likewise reduced the levels of statistical significance. Nevertheless, betel chewing continued to stand out as an important risk factor for male larynx cancer and oral cancer of both sexes. Likewise urban residence and *miang* chewing maintained their association with female lung cancer.

### *Smoke analysis*

Tar, nicotine and water values are given in Table IV and sample types are compared with Australian cigarettes on a unit weight basis in Tables V and VI. Although

TABLE II.—*Relative Risk Factors for Selected Variables, Adjusted for Age and Province of Residence*

| Risk variable | Oral and oropharynx: Male | Oral and oropharynx: Female | Larynx and hypopharynx: Male | Lung: Male | Lung: Female |
|---|---|---|---|---|---|
| Agricultural employment | 4·70** | 3·70* | 2·27* | 0·71 | 0·72 |
| Rural residence | 2·72* | 2·93* | 1·12 | 0·78 | 0·46* |
| School attendance | 0·23*** | 0·98 | 0·50* | 2·04* | 1·08 |
| Cigarette smoking | 0·39* | 0·18 | 0·74 | 1·75 | 2·08* |
| Pipe smoking | 0·79 | 12·27* | 1·07 | 0·12* | 0·00 |
| *Khii yoo* smoking | 0·61 | 1·06 | 1·17 | 1·77 | 1·71 |
| *Yaa muan* | 2·70 | 1·53 | 2·97* | 0·69 | 1·37 |
| Betel chewing | 2·94** | 3·21* | 2·71*** | 0·60 | 0·73 |
| *Miang* chewing | 0·77 | 0·76 | 2·23* | 1·06 | 2·02* |

* $P < 0{\cdot}05$.
** $P < 0{\cdot}01$.
*** $P < 0{\cdot}001$.

TABLE III.—*Multivariate Analysis of Relative Risks for Selected Factors, by Site and Sex*

| Site | Sex | Age† (45–64) | Age† (65 +) | Outside Chiang Mai province | School attendance | Cigarette smoking | *Khii yoo* smoking | *Yaa muan* smoking | Betel chewing | *Miang* chewing | Agri-cultural employ-ment | Rural residence |
|---|---|---|---|---|---|---|---|---|---|---|---|---|
| Oral and oropharynx | M | 0·95 | 1·04 | 1·27 | 0·32** | — | — | — | 2·27* | — | 2·09 | 1·80 |
| | F | 1·02 | 1·16 | 4·56** | 2·41 | — | — | — | 3·16* | — | 3·03 | 2·16 |
| Larynx and hypopharynx | M | 0·77 | 1·14 | 1·81* | 0·64 | — | — | 2·04 | 2·36** | 1·45 | 1·41 | 0·91 |
| Lung | M | 1·58 | 1·03 | 3·97** | 1·97* | 1·65 | 1·21 | — | — | 1·25 | — | 1·08 |
| | F | 0·73 | 0·35 | 0·71 | 0·94 | 1·63 | 1·47 | — | — | 2·23* | — | 0·42** |

* $P < 0{\cdot}05$.
** $P < 0{\cdot}01$.
† Relative to the under-45 age group.

TABLE IV.—*Tar, Nicotine and Water Values Obtained on Smoking Thai Cigars from Chiang Mai Province*

| | | | | Weight—mean (s.d.) | | | |
|---|---|---|---|---|---|---|---|
| Sample no. | Sample type | Length (cm) | Origin | Sample—g | Dry tar—mg | Nicotine—mg | Water—mg |
| 1 | *Khii yoo* (k) | 22 | Chiang Mai City | 7·51 (0·54) | 237 (35) | 14 (3) | 42 (24) |
| 2 | *Khii yoo* (k + t) | 19·5 | Chiang Mai City | 7·50 (0·64) | 302 (70) | 12 (2) | 83 (34) |
| 3 | *Khii yoo* (k) | 19 | Chiang Mai City | 8·09 (0·69) | 309 (143) | 8 (4) | 57 (46) |
| 4 | *Khii yoo* (k) | 19 | Chiang Mai City | 7·60 (0·91) | 211 (70) | 9 (2) | 38 (31) |
| 5 | *Khii yoo* (k = t) | 17 | Chiang Mai City | 6·01 (0·69) | 145 (136) | 6 (5) | 31 (39) |
| 6 | *Khii yoo* (t) | 13·5 | Chiang Mai City | 4·99 (0·35) | 149 (44) | 8 (3) | 23 (24) |
| 7 | *Khii yoo* (k) | 18·5 | County Suthep | 4·72 (0·67) | 207 (42) | 16 (3) | 58 (26) |
| 8 | *Khii yoo* (t) | 12 | County Suthep | 3·36 (0·44) | 159 (33) | 7 (2) | 62 (43) |
| 9 | *Khii yoo* (t) | 18 | County Suthep | 5·71 (0·76) | 226 (36) | 10 (2) | 50 (26) |
| 10 | *Khii yoo* (k) | 17 | County San | 4·11 (0·18) | 117 (39) | 8 (1) | 15 (9) |
| 11 | *Khii yoo* (k) | 18·5 | Nong Pa Krang | 4·50 (0·36) | 59 (26) | 6 (2) | 22 (19) |
| 12 | *Khii yoo* (k) | 14 | Nong Pa Krang | 3·26 (0·36) | 77 (20) | 9 (2) | 3 (2) |
| 13 | *Yaa muan** | 8 | Chiang Mai City | 1·42 (0·16) | 62 (11) | 6 (2) | 6 (2) |
| 14 | *Yaa muan** | 8 | Chiang Mai City | 1·41 (0·11) | 91 (7) | 9 (2) | 13 (4) |
| 15 | *Yaa muan* | 10·5 | County Suthep | 1·92 (0·24) | 140 (16) | 16 (3) | 15 (6) |
| 16 | *Yaa muan** | 8·5 | County Suthep | 1·39 (0·12) | 108 (23) | 10 (2) | 14 (14) |
| 17 | *Yaa muan* | 13·5 | County San | 1·95 (0·13) | 78 (12) | 7 (1) | 5 (2) |
| 18 | *Yaa muan* | 10 | Nong Pa Krang | 1·38 (0·18) | 79 (11) | 8 (1) | 5 (8) |
| 19 | *Yaa muan* | 8·5 | Nong Pa Krang | 1·47 (0·29) | 63 (23) | 7 (3) | 12 (12) |
| 20 | *Traa kai** | 7 | Chiang Mai City | 1·31 (0·05) | 44 (11) | 3 (3) | 5 (3) |
| 21 | *Traa kai** | 7 | Chiang Mai City | 1·28 (0·10) | 39 (7) | 3 (1) | 3 (3) |

k—*Khooi* bark.
*t*—tamarind fruit.
*—two cigars smoked per filter pad.

TABLE V.—*Average Dry Tar and Nicotine Content of the Mainstream Smoke from Thai Cigars*

| | | Dry tar | | Nicotine | |
|---|---|---|---|---|---|
| Sample type | Mean weight (g) | Total (mg) | mg/g cigar | Total (mg) | mg/g cigar |
| *Yaa muan* | 1·6 | 89 | 57 | 9 | 5·6 |
| *Khii yoo* | | | | | |
| Small | 3·3 | 77 | 23 | 9 | 2·7 |
| Medium (mean) | 5·6 | 183 | 33 | 9·5 | 1·8 |
| Large | 8·1 | 309 | 38 | 8 | 1·0 |
| *Traa kai* | 1·3 | 42 | 33 | 3 | 2·3 |
| Australian cigarettes | 1·0 | 5–20 | 5–20 | 0·3–1·3 | 0·3–1·3 |

the results set out in Table IV show a wide range of tar and nicotine values for samples of similar make, it is evident that there are marked differences between the three major types of cigars investigated.

TABLE VI.—*Nicotine Content of Australian and Indigenous Thai Tobaccos*

| Sample | Average nicotine (mg/g tobacco) |
|---|---|
| *Yaa muan* | 26 |
| *Traa kai* | 13 |
| *Khii yoo* | 15 |
| Australian tobaccos | 16–21 |

Table V shows that for each gram of cigar smoked, the mainstream smoke of *yaa muan* yields substantially higher quantities of tar and nicotine than the corresponding *khii yoo*. During collection of samples, it was observed (de Jong) that *khii yoo* cigars contain equal amounts of indigenous tobacco and additives. Our results (Table VI) indicate that they contain almost 50% less extractable nicotine and, on smoking, produce about half as much total particulate matter as the *yaa muan* cigars. This suggests that the individual additives of *khooi* bark and dried

TABLE VII.—*pH Analysis of Mainstream Smoke of Thai Cigars*

| Sample no. | Sample type | Additive* | pH range | Mean pH |
|---|---|---|---|---|
| 1 | *Khii yoo* | k | 5·1–5·3 | 5·2 |
| 2 | *Khii yoo* | k + t | 4·5–4·8 | 4·6 |
| 3 | *Khii yoo* | k | 4·9–5·3 | 5·1 |
| 4 | *Khii yoo* | k | 5·0–5·2 | 5·1 |
| 5 | *Khii yoo* | k + t | 4·9–5·2 | 5·0 |
| 6 | *Khii yoo* | t | 5·5–6·0 | 5·8 |
| 7 | *Khii yoo* | k | 5·2–5·7 | 5·5 |
| 8 | *Khii yoo* | t | 5·1–5·3 | 5·2 |
| 9 | *Khii yoo* | t | 4·8–5·4 | 5·1 |
| 10 | *Khii yoo* | k | 4·7–5·1 | 4·8 |
| 13 | *Yaa muan* | Nil | 7·9–8·8 | 8·3 |
| 14 | *Yaa muan* | Nil | 6·2–7·7 | Steady increase |
| 15 | *Yaa muan* | Nil | 7·8–8·5 | 8·0 |
| 16 | *Yaa muan* | Nil | 7·8–8·7 | 8·3 |
| 17 | *Yaa muan* | Nil | 6·4–6·8 | 6·5 |
| 18 | *Yaa muan* | Nil | 7·2–8·2 | 7·7 |
| 20 | *Traa kai* | Not known | 7·2–8·6 | Steady increase |
| 21 | *Traa kai* | Not known | 7·5–8·5 | 8·0 |

* k = *Khooi*, t = tamarind.

tamarind contribute little to the overall quantity of total particulate matter obtained for *khii yoo*.

Tamarind pods contain fairly high proportions of citric and tartaric acids. In the belief that these could have a marked effect on the pH of the smoke, we carried out pH analysis of the mainstream smoke of Thai samples (Table VII). The results show that all the *khii yoo* samples investigated produced a comparatively acid smoke, irrespective of the type of additive. Thus, both *khooi* bark and tamarind fruit additives appear to modify the *khii yoo* smoke to such an extent as to make it more acidic than, for example, Virginia-blended cigarette smoke (Sensabaugh and Cundiff, 1967; Hoffmann *et al.*, 1973). The *yaa muan* cigars, on the other hand, yield a relatively alkaline smoke, not unlike that of conventional European cigars.

### DISCUSSION

Several considerations complicate the interpretation of the results of hospital-based case-control studies such as this. Firstly, not all cases occurring in a defined geographic area or population are included in the study. This means that certain factors may operate to select those cases referred to a particular hospital which do not operate similarly on the controls and hence serve to confound the case-control comparison. In the present instance, it seems that cancer patients living outside Chiang Mai province were more likely to attend the university hospital than were patients with other diagnoses. However, the observed discrepancy could also result from true differences in incidence rates for the different provinces, which seems particularly plausible for female lung cancers. Unfortunately, without a population survey, there is no way to discriminate between these explanations.

Interpretation is further complicated by a high degree of correlation between proposed risk variables. In this study, oral and laryngeal groups of cancers were associated with a traditional rural agricultural life-style, of which betel chewing and *yaa muan* smoking, for example, are but two ingredients. Similarly, lung cancers in females were associated with *miang* chewing, cigarette and *khii yoo* smoking and urban residence. *Miang* chewing and *khii yoo* smoking were very closely related in this population, with 31% of *miang* chewers among control females reporting that they also smoked *khii yoo*, as opposed to only 9% of non-*miang* chewers. In fact 50% of *miang* chewers reported smoking (usually *khii yoo*) and chewing simultaneously. While the multivariate statistical procedures used attempt to adjust for the confounding effects of certain selection factors and the joint effects of correlated risk variables, such adjustments are limited by the amount of case material available, by the high degree of correlation between some factors, by memory bias, and perhaps by the failure to obtain data on relevant factors which are not yet even under suspicion. Thus, the identification of risk factors can be an uncertain process, especially in a single study.

*Oral cavity and oropharynx*

Evidence presented here for the dominant role of betel chewing in cancer of the oral cavity in both sexes confirms the findings of other studies. In contrast, the chewing of *miang* appears to have no effect. All those chewing betel nut reported they added lime and catechu wrapped together in a dried or fresh piper leaf; 25/26 betel-chewing cancer patients also added tobacco to the quid, whereas fewer than two thirds of the control chewers did so. Although these findings apparently favour tobacco as the major carcinogenic agent, other possibly related factors, such as other additives in the quid, dental sepsis (Shanta and Krishnamurthi, 1959) and nutritional deficiency (Wynder and Mabuchi, 1972) were not examined and cannot therefore be excluded. Although experimental evidence, reviewed by Muir and Kirk (1960) points to tobacco as the major causal factor in oral cancer, in New Guinea, oral cancer is frequent among coastal dwellers chewing betel nut with lime but without the addition of tobacco (Atkinson *et al.*, 1964). Smoking however is common.

We found no correspondence between the site where the quid was usually kept and the site of cancer. However, the numbers were rather small and, due to the advanced stage of the disease, it was often impossible to determine exactly the place of origin of the tumours within the oral cavity. About 20% of persons interviewed did not indicate a preferred site for keeping the quid.

With the exception of female pipe smoking, neither smoking nor alcohol consumption were related to oral cancer.

*Laryngeal region*

Although in males the relative risks associated with *yaa muan* and betel chewing were both about 2 when adjusted for each other (Table III), only the latter was statistically significant. The number of female cases was too small to draw valid conclusions. Jussawalla and Deshpande (1971) found that betel chewing along had a higher risk than smoking alone, for cancers of the oral cavity and hypopharynx, with increasing risk if the habits were combined. For oropharyngeal and laryngeal cancer, the pattern was reversed (*i.e.*, smoking alone carried a higher risk than chewing alone) with again an increased risk for combined habits. Similar associations may have occurred in the present series, and been obscured by our grouping of hypopharynx with larynx—necessary because when patients came in at a late stage of the disease it was very difficult to decide, for example, whether to classify tumours astride the aryepiglottic fold to the hypopharynx or to the larynx.

*Lung*

Cigarette and *khii yoo* smoking, while yielding risks of 1·7 to 2, were not strongly implicated for either sex. A larger study might show these trends to be statistically significant. In females, urban residence and *miang* chewing appeared to be the most important risk factors, both on a univariate and multivariate basis. The role of *miang* chewing must nevertheless be viewed with caution since the numbers on which it is based are small, no similar relationship was found in males, there is no plausible biological explanation, and many women chew *miang* and smoke *khii yoo* simultaneously. Thus, the possible aetiological role of *khii yoo* cannot be ruled out, especially in view of the laboratory results of acid smoke with high tar.

*Smoke analysis*

It seems that the nicotine and tar contents of the smoke of a *yaa muan* or *khii yoo* cigar may be more than 10 times as high as for an Australian cigarette (Table V). This difference is partly accounted for by the greater weight of the Thai cigars, but output per gram is also higher, especially for *yaa muan*. *Khii yoo* burn poorly and have to be frequently relit, possibly increasing tar yield (Wynder and Hoffmann, 1967).

The most important difference between *yaa muan* and *khii yoo* seems to be that in *khii yoo* the nature of the mainstream smoke is modified by the additives so as to make it lower in pH and more inhalable than *yaa muan* smoke. Hoffmann *et al.* (1973) proposed that pH and nicotine to a large extent determine the degree of smoke inhalability. An analytical comparison of cigarette and cigar smoke (Hoffmann and Wynder, 1972) has shown that the high nicotine concentration in cigar smoke coupled with high pH makes the inhalation of cigar smoke unpleasant as compared with cigarette smoke which is generally more acidic. Furthermore, the pH of mainstream smoke governs the degree of protonation of nicotine and hence its rate of absorption into the body (Armitage and Turner, 1970). Nicotine in alkaline smoke is absorbed chiefly through the lining of the mouth and pharynx, whilst nicotine in the acid smoke of cigarettes is absorbed chiefly after it has been inhaled into the lungs (Smoking and Health Now, 1971). *Khii yoo* smokers are apparently able to inhale the smoke very deeply without coughing (de Jong), suggesting that the mainstream smoke has greater inhalability than that of *yaa muan*: presumably because the former is relatively acid, whilst *yaa muan* smoke seems from our results (Table VII) to be relatively alkaline and therefore less likely to be inhaled deeply, although personal observation (de Jong) suggests that deep inhalation of *yaa muan* smoke is common among male smokers in Northern Thailand.

Although in our case-control study the associations of *yaa muan* and *khii yoo* smoking with laryngeal and lung cancers respectively were not statistically significant, the evidence that more *khii yoo* than *yaa muan* smoke may reach the lungs, and less remain in the upper respiratory tract, suggests that these associations may nevertheless be of aetiological importance, and that they should therefore be explored by further detailed studies of lifetime smoking history in suitable cases and controls.

We wish to thank Mrs Wimol Simarak and Mrs Nagnit Satapanakul for the persistent care with which the interviews were conducted, and Mr Donald Gibson in Chiang Mai and Miss Christine Mougne, for assistance with the classification of smoking habits. We are also grateful to Dr R. J. Mayfield and Mr D. R. Evans of C.S.I.R.O. for providing facilities for the pH measurements, to Miss Farideh Ghoddoussi and Mr T. E. Hassett for technical assistance, and to Dr C. S. Muir, IARC, and Miss Mougne for helpful critical comments.

Addendum: Since going to press we have become aware of the "Notification of the Royal Institute concerning the transcription of Thai characters into Roman (1941, J. Thailand Research Soc. **33**, part 1–2). Thus the cigars and additives should be spelled: *khi yo, buri ya muan, tra kai khoi* bark.

### REFERENCES

ANDERSON, J. A. (1973) Logistic Discrimination with Medical Applications. In *Discriminant Analysis and Applications*, Ed. Cacoullos, T. New York: Academic Press.

ARMITAGE, A. K. & TURNER, D. M. (1970) Absorption of Nicotine in Cigarette and Cigar Smoke through the Oral Mucosa. *Nature, Lond.*, **226**, 1231.

ATKINSON, L., CHESTER, I. C., SMYTH, F. G. & TEN SELDAM, R. E. J. (1964) Oral Cancer in New Guinea. *Cancer, N.Y.*, **17**, 1289.

BATES, W. W., GRIFFITH, R. B., HARLOW, E. S., SENKUS, M. & WAKEHAM, H. (1968) Determination and Reporting of Total Particulate Matter, Water in Total Particulate Matter, and Nicotine in Cigarette Smoke. *Tobacco Sci.*, **12**, 192.

HOFFMANN, D. & WYNDER, E. L. (1972) Smoke of Cigarettes and Little Cigars: an Analytical Comparison. *Science, N.Y.*, **178**, 1197.

HOFFMANN, D., RATHKAMP, G., BRUNNEMANN, K. D. & WYNDER, E. L. (1973) Chemical Studies on Tobacco Smoke XXII. *The Science of the Total Environment*, **2**, 157.

JACIN, H., SLANSKI, J. M. & MOSHY, R. J. (1968) The Determination of Nicotine in Tobacco and in Particulate Matter of Smoke by Gas Chromatography. *Anal. chim. Acta*, **41**, 347.

JUSSAWALLA, D. J. & DESHPANDE, V. A. (1971) Evaluation of Cancer Risk in Tobacco Chewers and Smokers: an Epidemiological Assessment. *Cancer, N.Y.*, **28**, 244.

MANTEL, N. & HAENSZEL, W. (1959) Statistical Aspects of the Analysis of Data from Retrospective Studies of Disease. *J. natn. Cancer Inst.*, **22**, 719.

MENAKANIT, W., MUIR, C. S. & JAIN, D. K. (1971) Cancer in Chiang Mai, Northern Thailand. A Relative Frequency Study. *Br. J. Cancer*, **25**, 225.

MUIR, C. S. & KIRK, R. (1960) Betel, Tobacco and Cancer of the Mouth. *Br. J. Cancer*, **14**, 597.

PILLSBURY, H. C., BRIGHT, C. C., O'CONNOR, K. J. & IRISH, F. W. (1969) Tar and Nicotine in Cigarette Smoke. *Jnl. Ass. Off Analyt. Chem.* **52**, 458.

PIYARATN, P. (1959) Relative Incidence of Malignant Neoplasms in Thailand. *Cancer, N.Y.*, **12**, 693.

PRENTICE, R. L. (1976) Use of the Logistic Model in Retrospective Studies. *Biometrics*, **32**, 599.

ROTHWELL, A. & GRANT, C. A. (1972) Standard Methods for the Analysis of Tobacco Smoke. *Tobacco Research Council Research Paper No.* 11, London.

SCHULTZ, F. J. & SPEARS, A. W. (1966) Determination of Moisture in Total Particulate Matter. *Tobacco Sci.*, **10**, 75.

SENSABAUGH, A. J. & CUNDIFF, R. H. (1967) A New Technique for Determining the pH of Whole Tobacco Smoke. *Tobacco Sci.*, **11**, 25.

SHANTA, V. & KRISHNAMURTHI, S. (1959) A Study of Aetiological Factors in Oral Squamous Cell Carcinoma. *Br. J. Cancer*, **13**, 381.

*Smoking and Health Now* (1971) A Report of the Royal College of physicians of London. London: Pitman Med. Sci. Pub. Co.

WYNDER, E. L. & HOFFMANN, D. (1967) Tobacco and Tobacco Smoke. *Studies in Experimental Carcinogenesis*. New York: Academic Press, Inc.

WYNDER, E. L. & MABUCHI, K. (1972) Etiological and Preventive Aspects of Human Cancer. *Prev. Med.*, **1**, 300.